# Intraoperative urinary tract resection and construction in CRS + HIPEC procedures: a single center retrospective analysis

**DOI:** 10.1186/s12957-024-03457-8

**Published:** 2024-06-26

**Authors:** Zhong-He Ji, Yu-Bin Fu, Gang Liu, Yang Yu, Bing Li, Yan-Dong Su, Rui Yang, Xin-Li Liang, Yan Li

**Affiliations:** 1 Department of Peritoneal Cancer Surgery, Beijing Shijitan Hospital, Capital Medical University, No. 10 Tieyi Road, Yangfangdian Street, Haidian District, Beijing, 100038 P. R. China; 2 Department of Surgical Oncology, Beijing Tsinghua Changgung Hospital Affliated to Tsinghua University, Beijing, 102218 China

**Keywords:** Cytoreductive surgery, Hyperthermic intraperitoneal chemotherapy, Urinary tract

## Abstract

**Introduction:**

The safety and efficacy of CRS + HIPEC combined with urinary tract resection and reconstruction are controversial. This study aims to summarize the clinicopathological features and to evaluate the safety and survival prognosis of CRS + HIPEC combined with urinary tract resection and reconstruction.

**Methods:**

The patients who underwent urinary tract resection and reconstruction as part of CRS surgery were retrospectively selected from our disease-specific database for analysis. The clinicopathological characteristics, treatment-related variables, perioperative adverse events (AEs), and survival outcomes were studied using a descriptive approach and the K-M analysis with log-rank comparison.

**Results:**

Forty-nine patients were enrolled. Perioperative serious AEs (SAEs) were observed in 11 patients (22.4%), with urinary SAEs occurring in 3 patients (6.1%). Additionally, there were 23 cases (46.8%) involving urinary adverse events (UAEs). The median overall survival (OS) in the entire cohort was 59.2 (95%CI: 42.1–76.4) months. The median OS of the UAE group and No-UAE group were 59.2 months (95%CI not reached), and 50.5 (95%CI: 11.5 to 89.6) months, respectively, with no significant difference (*P* = 0.475). Furthermore, there were no significant differences in OS based on the grade of UAEs or the number of UAEs (*P* = 0.562 and *P* = 0.622, respectively).

**Conclusion:**

The combination of CRS + HIPEC with urinary tract resection and reconstruction is associated with a high incidence of Grade I-II UAEs, which do not have an impact on OS. The safety profile of this combined technique is acceptable. However, this is a retrospective single-center single-arm analysis, with limitations of generalizability and potential selection bias. The findings need high-level validation.

**Supplementary Information:**

The online version contains supplementary material available at 10.1186/s12957-024-03457-8.

## Introduction

In the past four decades, the integrated treatment package comprising cytoreductive surgery (CRS) and hyperthermic intraperitoneal chemotherapy (HIPEC) has progressively gained recognition within the oncology community and become widely utilized for peritoneal surface malignancies [1–4].

The completeness of CRS is an independent determinant of the efficacy of this therapeutic [5]. In contrast to traditional debulking surgery, CRS is based on peritonectomy procedures developed by Sugarbaker’s team, which are used for resecting tumors on visceral intra-abdominal surfaces or stripping tumors from parietal peritoneal surfaces. The essence of CRS lies in the complete removal of macroscopic tumors through comprehensive resection of invaded peritoneum and multiple organs, thereby establishing a solid foundation for maximizing the efficacy of HIPEC [6].

Generally, the ureters are identified and preserved, when performing pelvic peritonectomy during CRS, followed by the en bloc removal of the entire pelvic peritoneum including the peritoneum of the posterior wall of the bladder and the peritoneal reflection with posterior pelvic organs. In cases where tumor invasion extends beneath the pelvic peritoneum involving the urinary tract, partial resection and reconstruction of the urinary tract may be necessary. Despite its potential benefits, combining CRS + HIPEC with urinary tract resection and reconstruction remains limited in clinical practice due to the procedural complexity, technical challenges, and surgical trauma. Thus, raising concerns regarding its safety and efficacy [7].

In the opposite opinion, CRS + HIPEC is still regarded as an aggressive solution for peritoneal metastasis, offering potential survival advantages alongside a notable risk of mortality and morbidity. When urinary tract is involved, even CRS + HIPEC is not recommended. Additionally, it is unequivocally contraindicated to perform CRS + HIPEC with urinary tract resection as it violates both surgical and oncological principles. Therefore, the practice of CRS + HIPEC involving urinary tract resection and construction is a topic of considerable controversy. There is an urgent need for comprehensive and systematic research in this area to address the existing knowledge gaps and uncertainties.

As a dedicated center specializing in peritoneal surface malignancies, our team has performed over 2000 CRS + HIPEC procedures and established a single-center disease-specific database. In this retrospective study, we analyzed the clinical data of the patients who underwent combined CRS + HIPEC with urinary tract resection and reconstruction for urinary tract invasion, aiming to summarize the clinicopathological characteristics and evaluate the safety and survival prognosis.

## Patients and methods

### Patients and database

The present retrospective analysis was conducted using our disease-specific database established at Beijing Shijitan Hospital, Capital Medical University. As of June 30, 2022, a total of 1961 cases were included in the database. Among these, 129 cases underwent urinary tract (ureter, bladder, urethra) resection and reconstruction as part of CRS surgery. Excluding 80 cases involving mere placement of ureteral stent, resulted in a final inclusion of 49 anal cases. The basic clinicopathological characteristics, details of CRS + HIPEC procedures, perioperative safety and survival outcomes were meticulously scrutinized to establish a comprehensive research database. This study followed the relevant provisions of the Declaration of Helsinki and was approved by the hospital Ethics Committee. All patients signed preoperative Institutional Review Board-approved consent.

### CRS + HIPEC

The standard CRS + HIPEC procedure was performed on all cases by a team of peritoneal oncologists in strict adherence to the Chinese expert consensus on CRS + HIPEC for peritoneal malignancies [3]. The Peritoneal Cancer Index (PCI) was utilized to assess tumor burden and dissemination. Following exploration, CRS was conducted with peritonectomy procedures to remove macroscopic lesions. Subsequently, the Completeness of Cytoreduction (CC) score was employed to evaluate the effectiveness of CRS.

HIPEC was performed following CRS using the open technique. The temperature was 43.0 ± 0.3 ℃, with a perfusion duration of 60 min, and a flow rate of 400 ml/min. A total volume of 3000 ml saline was used as the solvent for the drug regimen, which included: (1) cisplatin + docetaxel; (2) cisplatin + mitomycin C; (3) doxorubicin + ifosfamide; and (4) Other regimens.

The reconstruction or repair of the gastrointestinal tract and urinary tract was performed after HIPEC. Subsequently, an intraperitoneal chemotherapy pump and abdominal drainage tube were inserted, the abdomen was closed, and the patient was transferred back to the intensive care unit post-surgery.

### Urinary tract resection and reconstruction

**Fig. 1:**
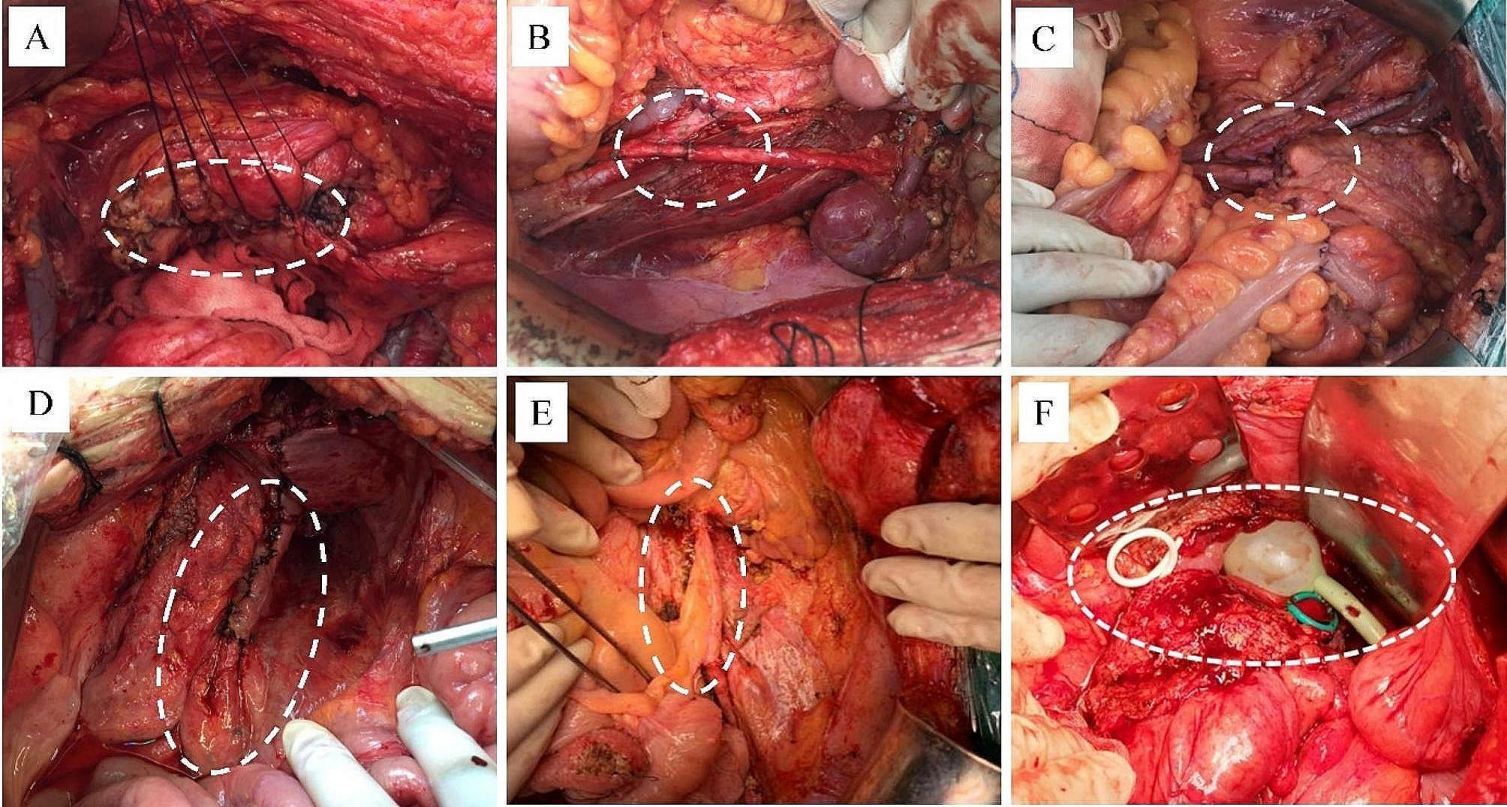
Technique of urinary tract reconstruction: bladder repair (**A**), ureteroureterostomy (**B**), cysto-urethral anastomosis (**C**), vesical flap-ureteral anastomosis (**D**), appendix orthotopic ureter creation (**E**), and implantation of the ureteral stent (**F**)

The resection procedures of the urinary tract investigated in this study encompassed radical cystectomy, partial cystectomy (excluding trigone cystectomy), partial ureterectomy, and combined partial cystectomy with partial ureterectomy. The technique of urinary tract reconstruction included bladder repair, ileal neobladder formation, cysto-urethral anastomosis, vesical flap-ureteral anastomosis, ureteroureterostomy, appendix orthotopic ureter creation, and implantation of the ureteral stent (Fig. 1). It is important to note that patients may undergo multiple types of urinary tract reconstruction.

### Perioperative adverse events (AEs)

Perioperative AEs were defined as AEs occurring within a 30-day postoperative period. The classification and grading of AEs followed the criteria recommended by the Peritoneal Surface Oncology Group International (PSOGI) [8]. Grade III–V AEs were defined as serious adverse events (SAEs).

### Follow-up

All patients were followed up after CRS + HIPEC through clinic and telephone. The follow-up appointments were scheduled every 3 months for the first 2 years post-surgery, every 6 months between 2- and 5-years post-surgery, and annually thereafter. Overall survival (OS) was defined as the time from the day of CRS + HIPEC surgery to death or the last follow-up.

### Statistical analysis

Descriptive statistics included frequency and percentage for categorical variables and mean ± standard deviation (SD) or median with range for continuous variables. The Kaplan–Meier method was used to describe survival and progression, and the log-rank test was used for comparison between the two groups. Univariate and multivariate Cox proportional model was conducted to identify the independent risk factors on OS. Statistical significance was defined as a p-value < 0.05. All data analyses were conducted using SPSS version 23.0 (IBM Corporation, Armonk, NY, USA). The cumulative survival curves were plotted using R version 3.5.1 (https://www.r-project.org/).

## Results

### Basic clinicopathological features

A total of 49 patients were enrolled in the study, with a median age of 52 (28–70) years old, comprising 41 females (83.7%) and 8 males (16.3%). The median Karnofsky Performance Status (KPS) score was 90 (60–100). There were 10 cases (20.4%) of retroperitoneal sarcoma, 8 cases (16.3%) of pseudomyxoma peritonei, and 5 cases (10.2%) from other sources. Forty patients (83.7%) had a history of abdominal surgery, 43 patients (87.8%) had a history of preoperative chemotherapy, and 7 patients (14.3%) had a history of preoperative radiotherapy (Table 1).

**Table 1:** Major clinicopathological characteristics of 49 patients treated with CRS + HIPEC

Characteristics	Value
Age (median, range) (yr)	52 (28–70)
Gender, n (%)	
Female	41 (83.7)
Male	8 (16.3)
Karnofsky Performance Status	
Median (range)	90 (60–100)
Primary tumor, n (%)	
Colorectal cancer	13 (26.5)
Ovarian cancer/fallopian tube cancer/primary peritoneal cancer	13 (26.5)
Retroperitoneal Sarcoma	10 (20.4)
Pseudomyxoma peritonei	8 (16.3)
Others	5 (10.2)
Abdominal surgery history, n (%)	
0	8 (16.3)
1	29 (59.2)
≥ 2	12 (24.5)
Preoperative chemotherapy, n (%) ^a^	
Yes	43 (87.8)
No	6 (14.3)
Preoperative radiotherapy, n (%)	
Yes	7 (14.3)
No	42 (85.7)

^a^ including intravenous, intraperitoneal chemotherapy and targeted therapy

### CRS + HIPEC related parameters

The median PCI score was 12 (1–39). Among the patients, 34 (69.4%) had a PCI < 20, while 15 patients (30.6%) had a PCI ≥ 20. The number of combined organ resection was < 4 in 33 cases (66.7%) and ≥ 4 in 16 cases (33.3%). 25 cases (51.0%) had 0–3 peritoneal resection areas, 18 cases (36.7%) had 4–6 peritoneal resection areas, and 6 cases (12.3%) had more than 6 peritoneal resection areas. Thirty-six patients (73.5%) achieved a CC0-1 cytoreduction, whereas thirteen patients (28.9%) obtained a CC2-3. HIPEC was performed on forty-four patients (89.8%) (Table 2).

**Table 2:** CRS + HIPEC related parameters of 49 patients treated with CRS + HIPEC

Characteristics	Value
Peritoneal cancer index	
Median (range)	12 (0–39)
< 20	34 (69.4)
≥ 20	15 (30.6)
Number of organ resection, n (%)	
0–3	33 (66.7)
≥ 4	16 (33.3)
Number of peritoneum resection, n (%)	
0–3	25 (51.0)
4–6	18 (36.7)
> 6	6 (12.3)
Completeness of cytoreduction, n (%)	
0–1	36 (73.5)
2–3	13 (28.9)
HIPEC, n (%)	
Yes	44 (89.8)
No	5 (10.2)
HIPEC regimens, n (%)	
CDDP + DOC	22 (50.0)
CDDP + MMC	7 (15.9)
DOX + IFO	4 (9.1)
Others	11 (22.4)
HIPEC duration, n (%)	
30 min	4 (8.2)
60 min	45 (91.8)
Fluid output volume at surgery (median, range)	
Blood loss (mL)	550 (100-6,000)
Urine output (mL)	1900 (400-5,850)
Ascites (mL)	0 (0–8,000)
Fluid infusion volume at surgery (median, range)	
RBC (U)	4 (0–20)
Plasma (mL)	800 (0–2,000)
Other fluid (mL)	6,475 (2,650 − 17,000)
Duration of CRS + HIPEC (min)	
Median (range)	665 (301-1,030)

HIPEC, hyperthermic intraperitoneal chemotherapy; CRS, cytoreductive surgery; CDDP, cisplatin; DOC, docetaxel; MMC, mitomycin C; DOX, doxorubicin; IFO, ifosfamide; RBC, red blood cells

### Urinary tract resection and reconstruction

**Fig. 2:**
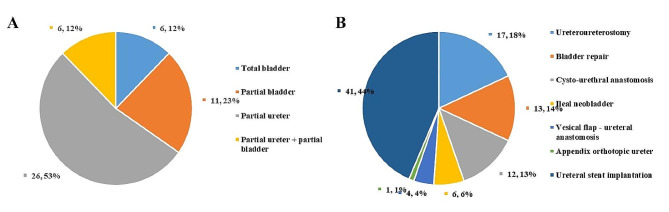
A pie chart of urinary tract resection (**A**) and reconstruction (**B**)

Total cystectomy was performed in 6 cases (12.2%), partial cystectomy in 11 cases (22.4%), partial ureterectomy in 26 cases (53.1%), partial cystectomy combined with partial ureterectomy in 6 cases (12.2%) (Fig. 2A). Various techniques were employed for urinary tract reconstruction, including bladder repair in 13 cases (26.5%), ileal neobladder in 6 cases (12.2%), cysto-urethral anastomosis in 12 cases (24.5%), vesical flap-ureteral anastomosis in 4 cases (8.2%), ureteroureterostomy in 17 cases (34.7%), appendix orthotopic ureter in one case (2.0%), and ureteral stent implantation in 41 cases (83.7%) (Fig. 2B). Among the patients, seven patients (14.3%) underwent one type of reconstruction, 38 patients (77.6%) underwent two types of reconstruction, and 4 patients (8.2%) underwent three types of reconstruction.

### AEs

**Fig. 3:**
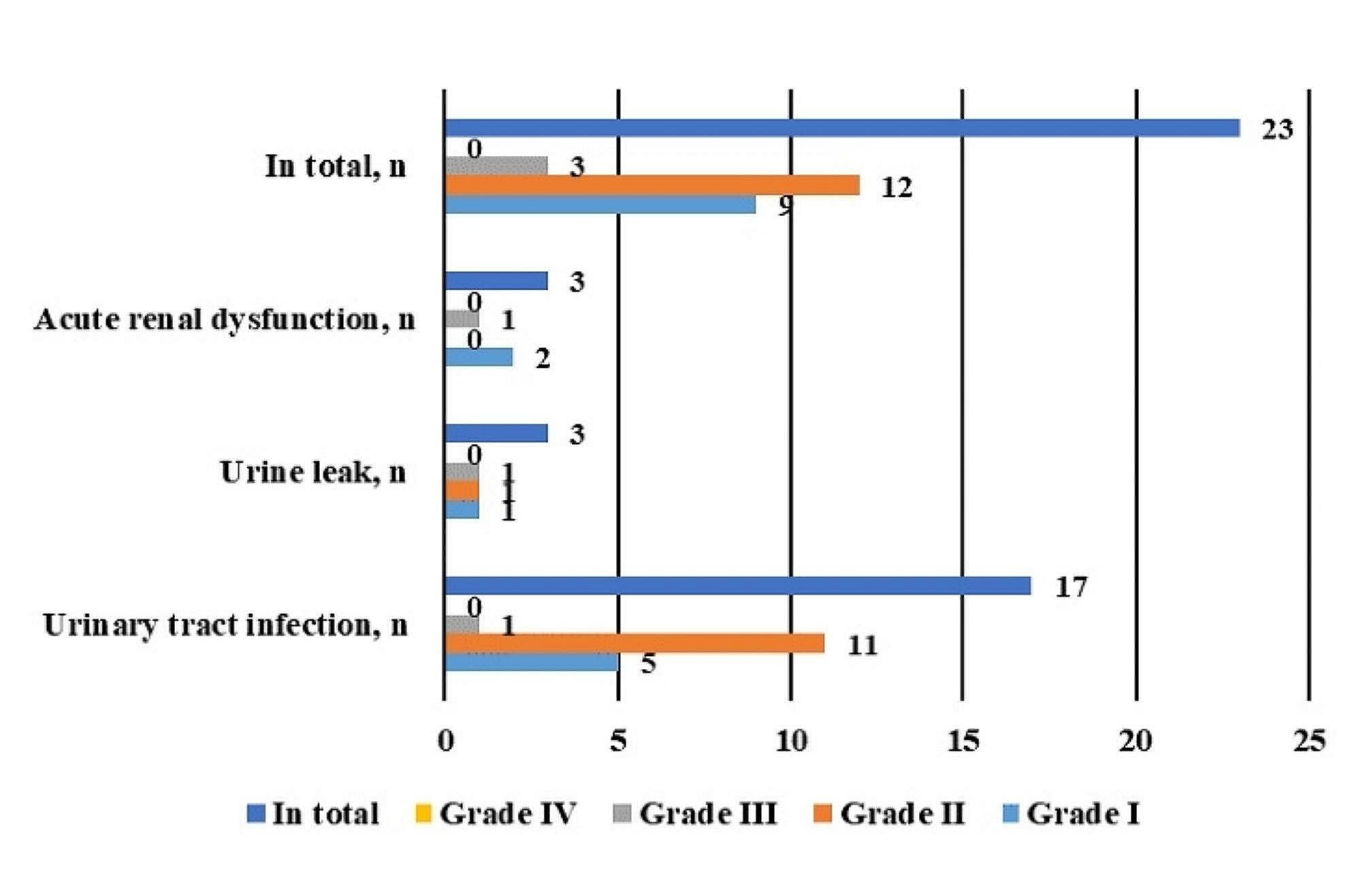
A bar graph of urinary system related adverse events

There were 11 cases of SAEs during the perioperative period, with an incidence rate of 22.4%. Among them, 3 cases (6.1%) were related to urinary system complications. Additionally, there were 23 cases (46.8%) involving urinary adverse events (UAEs), including 9 cases of grade I (16.3%), 12 cases of grade II (24.4%), 3 cases of grade III (6.1%), and 0 cases of grade IV (0.0%). The specific types of UAEs included urinary tract infection in 17 cases (34.6%), urinary leakage in 3 cases (6.1%), and acute renal insufficiency in 3 cases (6.1%) (Fig. 3). The median catheter indwelling time was 17 (0–86) days, the median ureteral stent indwelling time was 100 (30–370) days, and the median hospital stay was 27 (11–69) days.

### Survival analysis

**Fig. 4:**
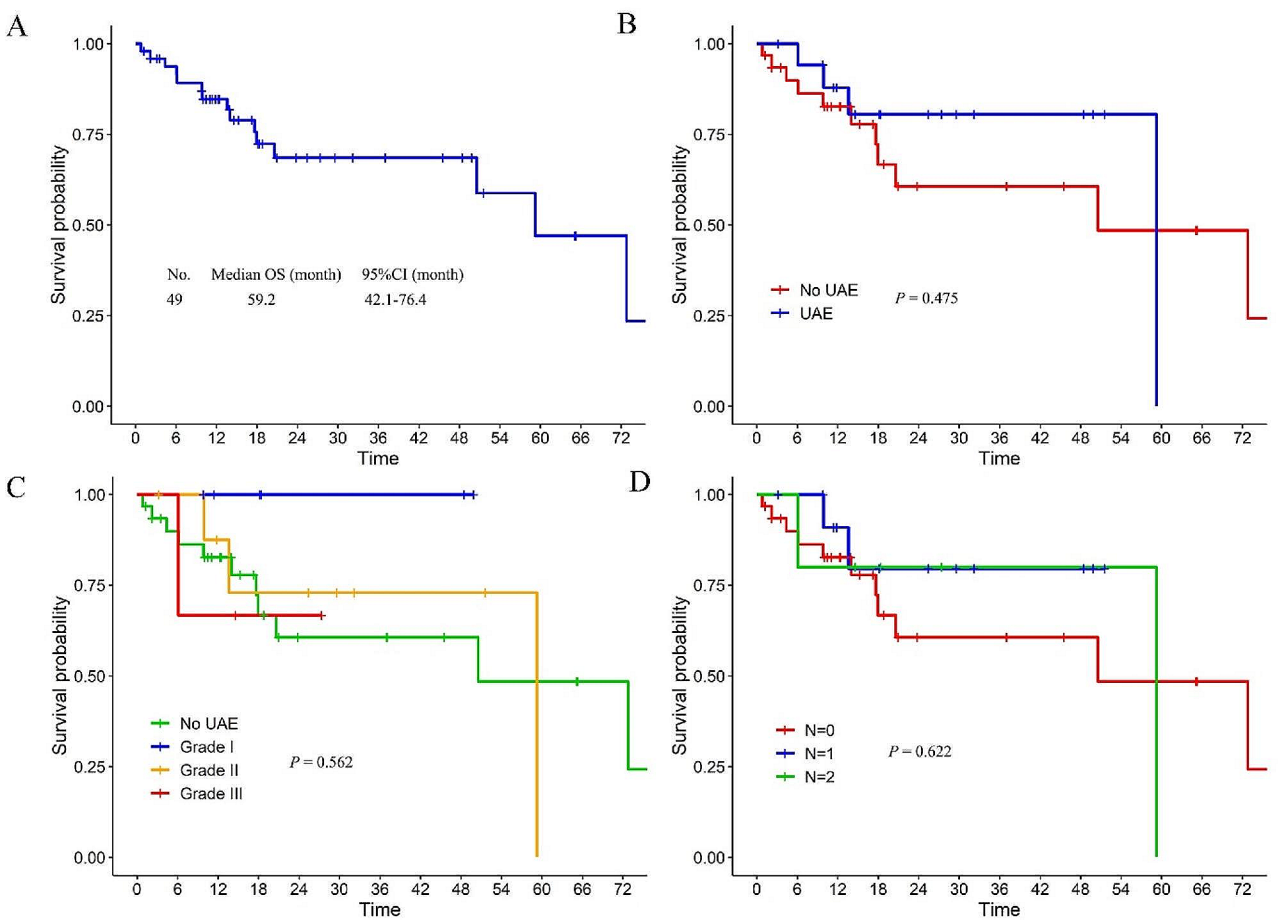
Kaplan–Meier analysis in the entire cohort (**A**), and log-rank comparison based on urinary adverse events (**B**), grade of UAEs (**C**), and number of UAEs (**D**)

The median OS in the entire cohort was 59.2 (95%CI: 42.1–76.4) months (Fig. 4A). The median OS of UAE group and No-UAE group were 59.2 months (95%CI not reached), and 50.5 (95%CI: 11.5 to 89.6) months, respectively, with no significant difference (*P* = 0.475) (Fig. 4B). Furthermore, there were no significant differences in OS based on the grade of UAEs (Fig. 4C) or the number of UAEs (Fig. 4D) (*P* = 0.562 and *P* = 0.622, respectively). The univariate analysis indicated that KPS (hazard ratio [HR] 0.941, 95% CI: 0.899–0.984, *P* = 0.008), PCI (HR 4.045, 95% CI: 1.392–11.748, *P* = 0.010), and CC (HR 11.085, 95% CI: 3.436–35.760, *P* < 0.001) were significantly associated with OS. In the subsequent multivariate analysis, CC (HR 9.858, 95% CI: 2.298–42.294, *P* = 0.002) emerged as the sole independent prognostic factor for OS (Supplement Table 1).

### Discuss

The combination of CRS + HIPEC with urinary tract resection and reconstruction remains limited in clinical practice, primarily due to its intricate operation process, technical complexity, surgical trauma, and uncertain survival benefit. The safety and efficacy of this approach continue to be a subject of controversy. In this retrospective analysis of a single-center cohort, we describe the fundamental clinicopathological characteristics, safety, and survival outcomes associated with CRS + HIPEC combined with urinary tract resection and reconstruction. Our findings reveal that the incidence of perioperative UAEs is notably high at 46.8%, although most are classified as grade I-II adverse events; importantly, these events do not significantly impact overall survival rates. Consequently, CRS + HIPEC combined with urinary tract resection and reconstruction represents a relatively safe alternative surgical strategy for patients presenting with peritoneal metastasis and invasion into the urinary tract.

CRS + HIPEC has garnered high-level evidence in the treatment of peritoneal malignancies and widespread recognized within the oncology community [9–15]. The technical core of CRS is the peritoneal resection technique proposed by Professor Paul H. Sugarbaker. This technical system removes macroscopic peritoneal metastatic lesions through combined organ resection to achieve complete tumor cytoreduction [6]. The techniques of peritoneal resection included anterior abdominal peritoneum resection, left upper abdominal peritoneum resection, right upper abdominal peritoneum resection, pelvic peritoneum resection, omentum and omental sac resection, and mesenteric peritoneum resection, comprising a total of six regions. In the process of pelvic peritoneal resection, middle and posterior pelvic organ resection and rectal anastomosis through an extraperitoneal approach have become well-established techniques. However, due to the special anatomical location, the pelvic peritoneum is often heavily invaded by peritoneal metastasis, frequently accompanied by invasion of the lower ureter and bladder. Total pelvic resection is a necessary measure to achieve regional radical resection in such patients. However, the surgical procedure of total pelvic resection is intricate, involving multi-system organ resection and functional reconstruction. It results in significant surgical trauma, high perioperative mortality and morbidity rates, a substantial impact on patients’ quality of life, and an uncertain prognosis [7, 16–18]. Consequently, in clinical practice, this radical surgical approach remains controversial and not yet widely implemented.

The technical safety and efficacy of CRS + HIPEC combined with urinary tract resection and reconstruction have been investigated in several domestic and international studies. Braam et al. [17] reported 38 patients with colorectal cancer peritoneal metastasis treated with CRS + HIPEC combined with urinary tract resection. The median survival time in this group was not found to be significantly different from that of the non-urinary tract operation group (26.9 months vs. 32.1 months, *P* = 0.29). However, the incidence of severe morbidity was significantly higher in comparison to the latter group (47% vs. 20%, *P* < 0.001). Morkavuk et al. [19] conducted a safety study on 20 cases of CRS + HIPEC combined with urinary tract resection and reconstruction, revealing that postoperative urinary tract complications occurred in 40% (8 cases). The occurrence of such complications was not associated with basic clinical characteristics including urinary tract reconstruction method, primary tumor, age, or gender, and did not significantly affect overall survival. However, the length of hospital stay nearly doubled after onset of disease (16.3 days vs. 8.8 days). Barrios et al. [20] reported the safety results of 14 cases of CRS + HIPEC + total pelvic exenteration, which underwent complex orthotopic bladder reconstruction without stoma during the operation. There were 5 cases (35.7%) of urinary leakage, including 3 cases classified as grade I-II, 1 case as grade III and 1 case as grade IV. Tuech et al. [7] reported 16 cases of peritoneal cancer patients treated with CRS + HIPEC + total pelvic resection, all achieving CC-0 resection. The incidence of grade III-IV morbidity was found to be 56.2%, and the perioperative mortality was 12.5%.

In our present study, there were 23 cases of UAEs in this study, with an incidence of 46.8%, including 9 cases of grade I (16.3%), 12 cases of grade II (24.4%), and 3 cases of grade III (6.1%). The overall incidence of UAEs in this study was consistent with the findings reported in previous studies. However, there were slight variations in specific manifestations. The most frequently encountered UAEs in this study were grade I-II urinary tract infections, while the incidence of severe urinary leakage and renal dysfunction was low.

Our study has several limitations. Firstly, it should be noted that this study is a retrospective single-center analysis, which inherently limits its generalizability due to the narrow focus on a single center’s experience. Secondly, the absence of a control group and reliance solely on historical data for comparison introduces certain methodological constraints. Lastly, potential selection bias may have influenced our findings as only a limited number of cases were included. We have merely presented our experience with CRS + HIPEC combined with urinary tract resection and construction, along with reporting the mortality, morbidity, and survival outcomes in this study. Therefore, it is crucial to further validate the conclusions drawn from this study through high-level evidence-based medicine. Following this descriptive study, we are now well-positioned to develop prospective control trials to investigate the safety and survival outcomes of CRS + HIPEC combined with urinary tract resection and reconstruction. We will establish patients with urinary tract invasion who underwent CRS + HIPEC without urinary tract resection and patients without urinary tract invasion who underwent CRS + HIPEC as control groups to compare safety profiles and survival outcomes with a non-inferiority objective, retrospectively.

## Conclusion

For patients with peritoneal malignancies, who are eligible for CRS + HIPEC and have a high potential for complete cytoreduction, the combination of CRS + HIPEC with urinary tract resection is a viable treatment option. However, it is important to note that the incidence of perioperative complications is high, and therefore clinical decision-making should be approached cautiously. Strict case screening should be implemented to avoid excessive aggressive treatment.

### Electronic supplementary material

Below is the link to the electronic supplementary material.

Supplementary Material 1

## Data Availability

The datasets used and/or analysed during the current study are available from the corresponding author on reasonable request.
