# Supplementary material for: Intraoperative urinary tract resection and construction in CRS + HIPEC procedures: a single center retrospective analysis
Source: World J Surg Oncol. 2024 Jun 26;22:171. doi: 10.1186/s12957-024-03457-8 (PMC11200841; doi:10.1186/s12957-024-03457-8)
Supplement: Supplementary file 1 — Supplementary Material 1 [file 12957_2024_3457_MOESM1_ESM.docx]

| Supplement Table 1. Univariate and multivariate analysis of risk factors for overall survival | | | | | | | |
| --- | --- | --- | --- | --- | --- | --- | --- |
| Factors | Univariate analysis | | |  | Multivariate analysis | | |
|  | HR | 95%CI | *P* |  | HR | 95%CI | *P* |
| Age (yr) | 1.009 | 0.958-1.062 | 0.742 |  |  |  |  |
| Gender (Male *vs*. Female) | 2.350 | 0.729-07.578 | 0.153 |  |  |  |  |
| KPS | 0.941 | 0.899-0.984 | **0.008** |  | 0.966 | 0.922-1.012 | 0.141 |
| Primary tumor, n (%) |  |  |  |  |  |  |  |
| Colorectal cancer *vs*. Others | 2.469 | 0.261-23.333 | 0.430 |  |  |  |  |
| Ovarian cancer/fallopian tube cancer/primary peritoneal cancer *vs*. Others | 0.880 | 0.089-8.736 | 0.913 |  |  |  |  |
| Retroperitoneal Sarcoma *vs*. Others | 2.280 | 0.228-22.804 | 0.483 |  |  |  |  |
| Pseudomyxoma peritonei *vs*. Others | 1.803 | 0.186-17.460 | 0.611 |  |  |  |  |
| PCI (≥20 *vs*. <20) | 4.045 | 1.392-11.748 | **0.010** |  | 1.237 | 0.299-5.126 | 0.769 |
| CC scores (CC2-3 *vs*. CC0-1) | 11.085 | 3.436-35.760 | **<0.01** |  | 9.858 | 2.298-42.294 | **0.002** |
| HIPEC regimen |  |  |  |  |  |  |  |
| CDDP+DOC *vs*. Others | 2.699 | 0.528-13.787 | 0.233 |  |  |  |  |
| CDDP+MMC *vs*. Others | 2.755 | 0.498-15.256 | 0.246 |  |  |  |  |
| DOX+IFO *vs*. Others | 3.903 | 0.322-47.290 | 0.285 |  |  |  |  |
| HIPEC duration (30 min *vs*. 60 min) | 0.034 | 0.000-26.654 | 0.320 |  |  |  |  |
| Number of organ resection (0-3 *vs*. ≥4) | 0.730 | 0.197-2.703 | 0.637 |  |  |  |  |
| Number of peritonectomy |  |  |  |  |  |  |  |
| 4-6 *vs*. 0-3 | 1.738 | 0.602-5.016 | 0.307 |  |  |  |  |
| >6 *vs*. 0-3 | - | - | - |  |  |  |  |
| SAE (Yes *vs*. No) | 2.049 | 0.684-6.141 | 0.200 |  | 1.157 | 0.334-4.007 | 0.818 |
| HR, hazard ratio; CI, confidence interval; KPS, Karnofsky performance score; PCI, peritoneal cancer index; CC, completeness of cytoreduction; CDDP, cisplatin; DOC, docetaxel; MMC, mitomycin C; DOX, doxorubicin; IFO, ifosfamide. | | | | | | | |
